# Electrophysiological and behavioral responses of elongated solifuge sensilla to mechanical stimuli

**DOI:** 10.1007/s00359-025-01731-y

**Published:** 2025-02-05

**Authors:** Pallabi Kundu, Mariela Oviedo-Diego, Franco Cargnelutti, R. Ryan Jones, Erika Garcia, Eileen A. Hebets, Douglas D. Gaffin

**Affiliations:** 1 School of Biological Sciences, University of Nebraska-Lincoln, Lincoln, USA; 2 Institute for Integrative Conservation, William & Mary, Williamsburg, VA United States; 3 Departamento de Diversidad Biológica y Ecología, Facultad de Ciencias Exactas, Físicas y Naturales, Universidad Nacional de Córdoba, Córdoba, Argentina; 4 Laboratorio de Biología Reproductiva y Evolución, Consejo Nacional de Investigaciones Científicas y Técnicas (CONICET), Instituto de Diversidad y Ecología Animal (IDEA), Córdoba, Argentina; 5 Department of Integrative Biology, University of Colorado, Denver, USA; 6 School of Biological Sciences, University of Oklahoma, Norman, USA

**Keywords:** Air particle movement, Nearfield sound, Mechanoreception, Camel spider, Predator avoidance, Sensory ecology

## Abstract

A fundamental understanding of animal sensory systems is crucial for comprehending their interactions with the environment and with other conspecifics. However, knowledge gaps persist, particularly in arachnids like the order Solifugae. While certain solifuge setae and palpal papillae have been studied structurally and electrophysiologically, providing evidence of chemoreception and mechanoreception, the sensilla on their walking legs remain unexplored. Notably, elongated sensilla on the femur and tibia of the 4th walking legs resemble trichobothria in other arachnid orders yet their function remains unknown. Thus, this study investigates whether these sensilla serve a mechanosensory function. Using electrophysiological and behavioral assays on *Eremobates pallipes* (Eremobatidae), we assessed the response of the elongated 4th leg sensilla to– (i) air particle movement and– (ii) air pressure changes. Air particle movement stimuli were generated using a speaker placed in the near field of the elongated sensilla that emitted low-frequency pure tones (10–1000 Hz). Air pressure stimuli involved forceful blowing on the sensilla. No response to air particle movement was observed, but a mechanosensory response to air pressure stimuli was detected. Electrophysiological data identified a fast-adapting and fast-recovering cell, and behavioral observations revealed a startle response. Our electrophysiology results suggest a mechanosensory role of elongated sensilla on the 4th walking legs of solifuge, indicating that although they are not sensitive enough to detect air particle movement stimuli, they can receive and respond to air pressure stimuli. Our behavioral experiments similarly show that these sensilla are not sensitive enough to detect air particle movement but respond to more forceful mechanosensory stimuli.

**Supplementary Information:**

The online version contains supplementary material available at 10.1007/s00359-025-01731-y.

## Introduction

Animals receive information from both their environment and other animals (e.g., conspecifics, heterospecifics, prey, predators) using a combination of sensory systems (Rowe 1999; Bradbury and Vehrencamp 2011; Hebets 2011; Higham and Hebets 2013). Specific sensory systems are often specialized for distinguishing stimuli of particular physical form - e.g., light, airborne chemicals, surface chemicals, pressure waves, etc. (Hebets and McGinley 2019). Reception of these stimuli provides animals with information that may be used in decision-making regarding foraging, mate finding, or that evoke anti-predator behavior, among others. Despite their importance, however, much remains unknown regarding the sensory capacities of numerous common, yet overlooked and understudied, organisms. Arachnids are one such group where fundamental revelations regarding sensory capabilities await discovery.

There are well over 110,000 species of arachnids (class Arachnida) (Agnarsson 2023) encompassing animals with diverse lifestyles (e.g., free-living, ectoparasites), living in distinct environments (e.g., leaf-litter; underwater; aerial), and engaging in often-unique behavior (e.g., net-casting; spitting silk/glue) (Yeargan 1975, 1994; Alberti and Zeck-Kapp 1986; Hebets and Chapman 2000; Beccaloni 2009; Stafstrom et al. 2020). Consequently, arachnids possess diverse and often taxon-specific sensory structures - e.g., elongate antenniform legs of amblypygids (Santer and Hebets 2011a), enlarged anterior median eyes of jumping spiders (Harland and Jackson 2002), pectines of scorpions (Foelix and Müller-Vorholt 1983; Gaffin and Brownell 1997a; Wolf 2017), slit sensilla or lyriform organ in various arachnids (Pringle 1955; Brownell 1977; Barth and Bohnenberger 1978; Brownell and Farley 1979; Young et al. 2016) and malleoli of solifuges (Brownell and Farley 1974), among others. While the sensory structures in many arachnids have been studied, ongoing investigations continue to uncover new functions. For example, amblypygid antenniform legs are not only sensory structures capable of receiving olfactory (Hebets and Chapman 2000; Hebets et al. 2014) and tactile information (Santer and Hebets 2011b), but they also produce air particle displacement, or air-borne vibrations, used for communication during agonistic interactions (Fowler-Finn and Hebets 2006; Santer and Hebets 2008, 2011a). Net-casting spiders can ‘hear’ airborne vibrations of flying insects from up to 2 m away using vibration sensors on the tips of their legs (Stafstrom et al. 2020); the orb-weaving spider *Argiope bruennichi* was recently shown to smell with sensilla on their legs (Talukder et al. 2025); and the antero-lateral eyes of jumping spiders help their principal eyes track moving prey, increasing the precision of the principal eye (Jakob et al. 2018). These are all examples of novel functions discovered in previously studied sensory structures. Many arachnid groups, however, possess a variety of distinct sensory structures whose functions remain unknown. In particular, many of the multiple types of arachnid sensilla have unconfirmed functions.

Arachnid sensilla are often mechanosensory and/or chemosensory, with numerous examples coming from research on *Cupiennius salei* (Barth 2000). Mechanoreception in particular is well documented in trichobothria and lyriform organs (Barth 2004) and has more recently been shown to be important in communications (Rundus et al. 2010; Choi et al. 2019; Kundu et al. 2022). Spiders also use mechanoreception in prey capture (Klärner and Barth 1982; Barth et al. 1995; Friedel and Barth 1997) and predator avoidance (Suter 2003) as well as for finding mates (Gibson and Uetz 2008; Kundu et al. 2022). Given the demonstrated importance of mechanoreception in the lives of numerous spiders (Foelix and Chu-Wang 1973; Barth 2000, 2004; Suter 2003) and other arachnid orders (amblypygids Fowler-Finn and Hebets 2006; Santer and Hebets 2008, 2011a), we hypothesize that the extraordinarily long sensilla on an understudied relative - Order Solifugae - also has a mechanosensory function. Given their length and superficial similarity with trichobothria, we hypothesize that these hairs may even function to detect air particle movement, or near-field sound (Rundus et al. 2010; Choi et al. 2019; Kundu et al. 2022).

Solifuges are a fascinating group of arachnids that vary in appearance, particularly related to body size and “hairiness” (Hewitt 1935; Punzo 1998; reviewed in Hebets et al. 2023). Over 1,200 species of solifuges have been identified worldwide and they are found predominantly in xeric environments on all continents except Antarctica and Australia (Schmidt 1993; Beron 2018; reviewed in Hebets et al. 2023). While these strong-jawed and fast-moving predators (Linsenmair 1968; Wharton 1986; Punzo 1998; reviewed in Hebets et al. 2023) are adept at both catching prey and escaping predators (Punzo 1998; Wharton and Reddick 2014; reviewed in Hebets et al. 2023), little is known about how solifuges acquire, process, and respond to environmental information. Sensorially, solifuges are best known for their malleoli, or racquet organs (Brownell and Farley 1974) which are present on the 4th pair of walking legs and function in detecting chemical signals (Brownell and Farley 1974; Sombke et al. 2019). Despite often being covered with sensilla of different forms, lengths, and presumably, function (Cushing and Casto 2012), few studies have explored these sensilla. Only one study to date has examined the form and function of the sensilla (termed setae/papillae) on solifuge pedipalps. The distal pores on the setae suggested a function in chemoreception (Cushing and Casto 2012). Transmission electron microscopy (for structure) followed by electrophysiology (for function) confirmed a mechanosensory function of these papillae (Cushing et al. 2014) with a possibility of chemoreception. The numerous additional sensilla on the walking legs of solifuges remain unstudied, including the extraordinarily elongated sensilla that are the focus of this study.

**Fig. 1:**
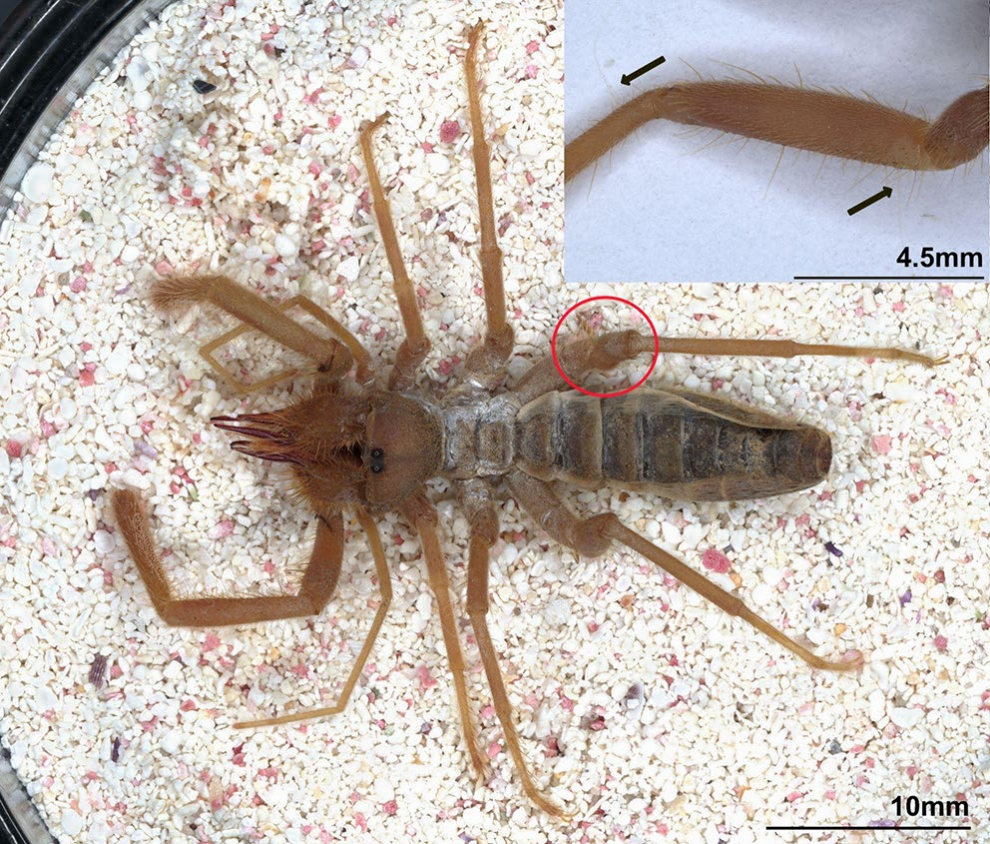
Solifuge (*Eremobates pallipes*) (arrows point to two elongated sensilla on femur and tibia of right 4th walking leg)

The extremely elongated sensilla on the 4th walking legs of some solifuges (Fig. 1) superficially resemble the trichobothria sensory sensilla found in most other arachnid orders. Consequently, some authors have cited the long hairs of solifuges as trichobothria (Cloudsley-Thompson 1961; Shultz 1990; Selden and Shear 1996). Although these sensilla feature a socketed base, they differ from true trichobothria due to the lack of a distinct bothrium (cup-shaped cuticular structure), which makes them structurally different (Shultz 1990; Barth and Holler 1999). The absence of trichobothria is shared with arachnid orders Opiliones and Ricinulei (Reissland and Görner 1985), as well as Parasitiformes (Lindquist 1984). Although the relationship of Solifugae to other arachnid orders has remained ambiguous, the majority of more recent studies leveraging genetic/genomic data indicate a sibling relationship with Acariformes (Alberti and Peretti 2002; Pepato et al. 2010; Dabert et al. 2010; Sharma et al. 2014; Ballesteros et al. 2022); a group that notably possesses trichobothria. Given the lack of robust phylogenetic placement for Solifugae, it is unclear if these elongated sensilla represent modified, homologous, or analogous structures relative to trichobothria. Further, since these sensilla have thus far avoided physiological investigation, no functional, anatomical, or physiological data exist to inform hypotheses on their evolutionary relationship to trichobothria. Consequently, establishing even a basic/fundamental understanding of solifuge sensory ecology will greatly expand the breadth and depth of future behavioral, ecological, and evolutionary understanding.

We are not the first to be intrigued by the extraordinarily long leg sensilla of solifuges, which can be more than twice as long as the trichobothria in other arachnids with respect to body length (Barth et al. 1993; Weygoldt 2000). Numerous early authors noted their abundance (Bernard 1896; Barrows 1925; Hilton 1932) and a potential function in prey capture was first explored by Bolwig in the 1950s (1952). He found no effect of removing the elongated sensilla from the pedipalps, but the removal of the elongated sensilla from the first leg or the entire body reduced foraging capacities (Bolwig 1952). In 1968, solifuges were again noted to possess long, fine sensilla sensitive to the slightest touch, but their function was, and still is, poorly understood (Grasse 1968). Conversely, the trichobothria of spiders are well known to facilitate the detection of air particle movement (Barth 2002), which is important in environmental interactions such as predator avoidance (Suter 2003) and prey capture (Friedel and Barth 1997), among others. Similar to spiders, solifuges are also both predators and prey (e.g., to birds, bats, etc.; Arlettaz et al. 1995; Anderson et al. 1999; Catenazzi et al. 2009) and could thus also benefit from detecting air particle movement. The structural similarity of the elongated sensilla to that of trichobothria as well as their similar predator/prey lifestyle lead us to ask the following question: can these elongated sensilla also detect air particle movement (i.e. movement of air particles in the near field of the animal)? To answer our question, we employed both electrophysiological and behavioral approaches.

Electrophysiological analyses have been very useful in understanding the biology of signal reception by arachnid sensory structures. The dorsal tarsal organs of sand scorpions (*Smeringurus mesaensis*), for example, were shown to be sensitive to humid air stimuli by electrophysiology (Gaffin et al. 1992). Electrophysiological studies have also revealed synaptic interactions between sensory neurons in peg sensilla on the pectines of scorpions (*S. mesaensis*, *Hadrurus arizonensis*, *Centruroides vittatus*), which are likely involved in the processing of chemical information (Gaffin and Brownell 1997a, b; Gaffin 2002; Gaffin and Shakir 2021). Furthermore, while performing electrophysiological studies on the tarsi of antenniform legs of an amblypygid (*Phrynus pseudoparvulus*), it was found that sensilla on these legs were responsive to a vast number of chemicals, confirming an olfactory function (Hebets and Chapman 2000). Building from these arachnid electrophysiology studies, we adapted similar techniques to broaden our understanding of the sensory function of the elongated sensilla on the 4th walking legs of solifuges. Using electrophysiology in addition to behavioral observations, we explored the sensitivity of these sensilla to two different types of mechanosensory stimuli - (i) air-particle movement and (ii) air pressure. There has been no previous study on the elongated sensilla on the hindlegs of solifuges, especially using electrophysiology in combination with behavioral tests, thus making this a novel endeavor.

## Methods

### Study animals

For the electrophysiology studies, we collected 12 solifuges (*Eremobates pallipes*) near the visitor center of the Rocky Mountain Arsenal National Wildlife Refuge, Colorado, USA (39°50’4.23” N, 104°50’21.52” W) between July 24–26, 2021. We initially housed each individual in a small Tupperware container with paper towel bedding that we replaced every day. Solifuges were exposed to natural day/night cycles, and we fed them 1–2 small live crickets (purchased from PetSmart) daily. Immediately prior to the electrophysiology experiments, we sent the solifuges overnight to the University of Oklahoma, Norman, OK, USA. Animals arrived on August 3, 2021, and we conducted experiments from August 4–8, 2021. During this time, we housed the solifuges in small plastic containers (diameter: 11 cm, height: 10 cm) with mesh lids. We filled the containers about halfway with sand and added a few small rocks. We fed each solifuge one ~ 1.3 cm live cricket (*Gryllodes sigillatus* from Ghann’s Cricket Farm) each day. Solifuges have poor survival in captivity (Cushing et al. 2014) and when the animals naturally expired, we put them in 70% ethanol. We retained the preserved specimens in the collection of the Hebets’ laboratory at the University of Nebraska-Lincoln following laboratory policy in the absence of any national or University-wide regulations for working with arachnids.

We used extracellular electrophysiology to record the electrical signature of cells at the base of individual sensilla and the responses of the solifuges to various stimuli. Out of the 12 available animals, we collected reliable data for air particle movement and air pressure stimuli from five (Table 1). We have shown two sets of representative data for each experiment in this study. As electrical signals vary amongst different cells of an animal and between animals, we have not combined the recording data from different animals or different sensilla.

**Table 1:** Biology of solifuges used in electrophysiological recordings and their recording details. The table contains Animal ID, sex, weight (mg), and cephalothorax width (mm) along with the type of stimuli used, # of recordings, and in which figures the data are visualized. The acronyms used for stimuli are APM (Air Particle Movement stimuli), AP (Air Pressure stimuli), and PB (Passive Breath stimuli). The acronyms used for the location of the sensilla on the 4th pair of legs are LT (Left Tibia), RT (Right Tibia), LF (Left Femur), and RF (Right Femur)

Animal ID	Sex	Weight (mg)	Cephalo- thorax width (mm)	Type of Stimuli	Location of hair	# of recordings	Data
A	Male	110	3.64	APM	LT	2	Figure 2
B	Female (gravid)	700	5.49	APM	LT	1	SI. 7
					RT	1	Data not shown
				AP	LT	3	Figure 3; SI. 9 A
					RT	1	SI. 8, 9B
				PB	RT	3	Figure 4; SI. 10
C	Male	480	4.74	APM	LF	1	Data not shown
				AP	LF	3	
D	Female (gravid)	600	6.47	APM	RF	1	Data not shown
E	Female	560	5.29	AP	LT	1	Data not shown

*Note*: Not all recording data is visualized in the figures

To analyze the elongated sensilla deflection and behavioral responses to different stimuli, we collected two solifuges (Eremobatidae, species unknown due to immaturity), from Cedar Point Biological Station in Ogallala, Nebraska, USA (41°12’30.7"N, 101°38’45.7"W, 967 masl) between July 20 - August 5, 2022, using pitfall traps with bright lights (Cushing and González-Santillán 2018). We note that these immature solifuges did not display notable differences in their elongated sensilla as compared to the mature *Eremobates pallipes* used in the electrophysiology studies. We used these animals to observe (i) elongated sensilla deflections with a high-speed video camera and (ii) individual behavioral responses to stimuli. We conducted these observations at Cedar Point Biological Station immediately following the capture of the solifuges. After the experiments, we placed the individuals in 70% alcohol and deposited them in the collection of the Hebets’ laboratory at the University of Nebraska-Lincoln.

### Elongate sensilla deflection

To examine the potential mechanical response of the elongated sensilla on the 4th leg to different frequencies of air particle movement, we subjected each solifuge to a cooling period in a glass vial for 3–5 min by placing them in a refrigerator (-20ºC). Once the solifuge was immobilized, we positioned it on a microscope slide with modeling clay to secure and immobilize the animal while exposing its left 4th leg. Subsequently, we placed the solifuge on a granite surface to prevent the transmission of surface-borne vibrations. The stimuli were produced following the same methodology as the electrophysiology study. We employed a high-speed camera (Photron Fastcam1024PCI, Model: 100 K) to observe the sensilla movement in real-time with a computer set-up. We recorded the movement of the sensilla with the camera operating at 5000 fps with a shutter speed of 1/5000 s in all the experimental frequencies (10–1000 Hz).

*Air particle movement stimuli*. The speaker was positioned 10 cm from the individual, and we played progressively increasing frequencies 10 to 1000 Hz (at 10 Hz increments until 300 Hz and then at 50 Hz increments to 1000 Hz) at different amplitudes (low: 70–80 dB, medium: 81–96 dB, high: 100–105 dB). Moreover, we played each frequency for 3 s and observed whether the elongated sensilla exhibited any movement.

*Air pressure stimuli*. We (author FC) applied “forceful breaths” 20 cm from the immobilized individual narrowing our lips to almost a point and letting out a short burst of air (less than one second) at different intervals (following the air pressure stimuli protocol in the Electrophysiology study) to observe the potential deflection of the elongated sensilla to a single prolonged burst lasting 5–6 s. In addition, we generated a “forced airflow” with a syringe (by activating the syringe of 20 cc and pressing gently for a duration of 5–6 s) also 20 cm from the immobilized individual.

### Electrophysiology of sensilla

#### Experimental setup

The experimental setup was arranged inside a Faraday cage placed on a vibration-dampening table (Technical Manufacturing Corporation, Peabody, MA, USA, SI. 1a). We placed a dissecting stereoscope (Nikon Olympus, 105478) inside the cage, with a micromanipulator (World Precision Instruments, M3301L) to hold the animal on the left side and another micromanipulator (Leitz, 100894) to position the electrode on the right side (SI. 1a). We used an amplifier (World Precision Instruments, DAM 80) to amplify the signals 1000 times over a bandwidth of 300–3000 Hz, displayed them on an oscilloscope (Tektronix, 5A18N), and relayed the signals through an analog-to-digital converter (CED, Micro3 1401) to a computer. We recorded all electrophysiological responses using Spike2 version 8 (CED).

To prepare a solifuge for electrophysiology, we put the animal in the freezer (~ -17.8ºC) in a glass vial for 3–5 min so that it became immobile. Following inactivity, we placed the animal on a slide with double-sided tape to affix its abdomen and fourth pair of legs (SI. 1b). Then, we used modeling clay to keep the front of the body in place and prevent escape (SI. 1b). We used additional double-sided tape as needed to restrain the animal from moving its legs during the experiment.

We affixed the slide with the solifuge to the first micromanipulator. We positioned the slide under the dissecting stereoscope inside the Faraday cage and focused the stereoscope on the base of an elongated sensilla on the femur and tibia of the left and right fourth pair of walking legs of the solifuges (Fig. 1). The sensilla were ~ 2.7–5.0 mm in length. We attached a tungsten recording electrode (that was electrolytically sharpened in a 1 M NaNO3 solution) to the Leitz micromanipulator. While observing the base of the sensillum under the stereoscope, we manipulated the recording electrode using the micromanipulator and pierced the base of the sensillum (SI. 1c). We also inserted a small piece of silver wire through the cuticle on the femur of either the left or right third walking leg to serve as the indifferent electrode to complete the circuit. We used the oscilloscope, the Spike2 computer record, and the sound of the signal (using a PYLE PTA2 Stereo Power Amplifier attached to a pair of small Realistic speakers) to search for consistent spikes (i.e. action potentials), which let us determine if the electrode was detecting extracellular neural activity from sensillar neurons. After the recordings were made, we released the animals back into their containers. All experimental animals were active and foraging after they were released.

#### Experimental stimuli

*Air particle movement stimuli.* We used speakers to introduce an air particle movement stimulus (i.e. near field sound) to deflect the elongated sensilla. The near field is determined as the area closest to the source of the sound, generally within less than one wavelength of the sound frequency. With the inverse relation between wavelength and frequency, and knowledge of the speed of sound (~ 34000 cm/s), we determined the wavelength of the frequencies that we used (10–1000 Hz). The wavelength of 10 Hz is ~ 3400 cm while the wavelength of 1000 Hz is ~ 34 cm. Thus, we placed our speaker (DD Audio, DB65A) ~ 10 cm away from the elongated sensilla ensuring near-field range for all frequencies used. We used an amplifier (Rolls, PA71plus MicroMix Power Amplifier) to connect the speaker to a laptop to play the sound files at 74dB, measured using a digital sound level (Brand: RadioShack, Model: Digital Sound Level Meter 33-2055). For the first animal (Table 1, Animal A), we used sound frequencies of 10 to 1000 Hz (at 10 Hz increments until 300 Hz and then at 50 Hz increments to 1000 Hz) to observe any response to air particle movement. In between each sound frequency stimulus, there was a gap of one to two minutes where no frequencies were played. These parts of the recordings were referred to as the “control”. In total, 4 of our solifuges produced a total of six clear sets of recordings during our air particle movement stimulations (Table 1). We also performed a preliminary behavioral trial (SI. 2) with similar parameters.

*Air pressure stimuli*. We (author PK) used “forceful breaths” to introduce an air pressure mechanosensory stimulus to deflect the focal elongated sensilla (SI. 3). Our choice of using “forceful breaths’’ was driven by reliable responses observed in our preliminary tests and the need to conduct experiments quickly before the animals expired. Time pressure and fragility of these understudied arachnids severely limited our experimental options and forced us to be creative with air pressure stimuli. To provide comparable stimuli across deliveries, a single experimenter (PK) repeatedly narrowed their lips to almost a point and let out a short burst of air (less than one second) at different intervals. We (author PK) also introduced a prolonged burst lasting 5–6 seconds. Given that the focus of this study was on the potential to detect stimuli and not on the relationship between stimulus characteristics and response, we only needed to be sure that our stimulus was indeed capable of stimulating our focal sensilla.

To ensure that our “forceful breaths” successfully moved the target sensillum, we captured a video (SI. 3) of the sensillum during our forceful breath stimuli and used a Python script to capture the deflection of the sensillum (see SI. 4). By using a frame-by-frame subtraction method coupled with a contour generating algorithm, we documented the moving sensillum within a cropped area of the video and visually represented the angle of deflection within each frame (see SI. 4). Finally, we generated a graph showing the angle of sensillum deflection for 10 stimuli extracted from the video (see SI. 5).

For some sensilla, we tested the presence/absence of responses to the forceful breath stimuli (Table 1, Animal B, Animal C, Animal E). For one animal (Table 1, Animal B), we systematically tested the response to forceful breath stimuli. For Animal B, we introduced the short bursts at intervals of three seconds (3s), two seconds (2s), and one second (1s). We introduced all stimuli from ~ 23 cm away. A complete set of air pressure stimuli included multiple short bursts of forceful breath (10–12) at 3s, 2s, and 1s intervals. In between the stimuli with different intervals, we administered rest periods of 15–30 s. We also performed a preliminary behavioral trial (SI. 6) with similar parameters.

We used prolonged forceful breath stimuli to determine the recovery of the cells. We collected three sets of systematic recordings of responses to forceful breath stimuli from two separate elongated sensilla in one solifuge (Table 1, Animal B). Since our breath stimuli also included humidity, temperature, and/or olfactory stimuli, we conducted a second stimulus set of ‘passive breaths’ to separate out the effects of those factors. This also acted as a control to the air pressure stimuli. For the passive breath stimuli, we opened our mouths wide and let out air in short bursts of around one second from ~ 23 cm away. We obtained three sets of recordings with the passive breath stimuli from one solifuge (Table 1, Animal B).

#### Analysis of electrophysiology recordings

**Fig. 2:**
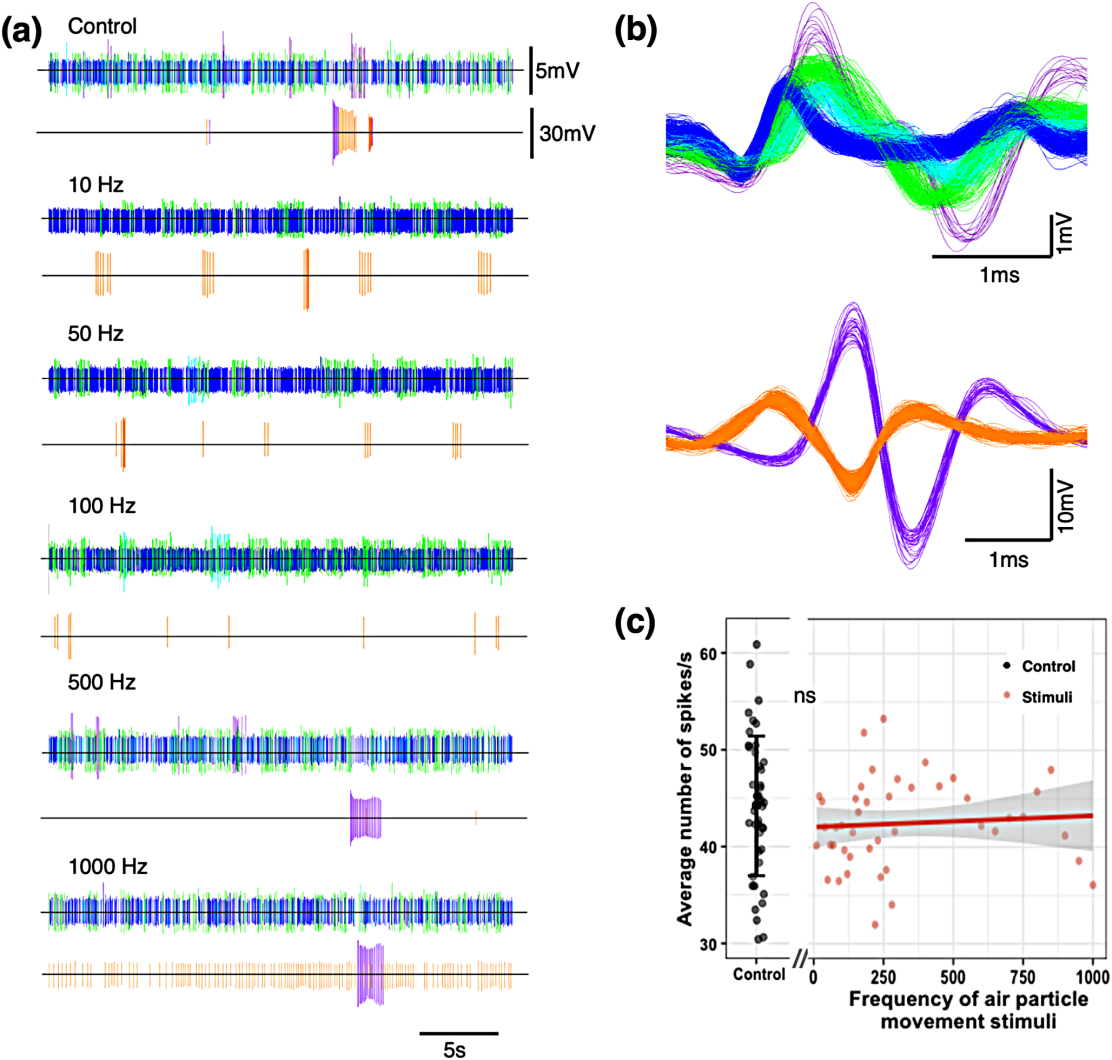
Lack of response to air particle movement stimuli (Animal A, Sensillum location: Left Tibia). (**a**) Representative electrophysiological recordings from a long sensory hair on the tibia of the left 4th leg at control (no stimulus), 10 Hz, 50 Hz, 100 Hz, 500 Hz, and 1000 Hz (stimuli). The upper traces are the sorted shorter spikes, and the bottom traces are the sorted taller spikes for each. Notice that there are no significantly different spikes appearing between the control and stimulus and the difference in axes scales. (**b**) The shorter spikes (Voltage: -3/+3.5 mV) are superimposed on the top and the taller spikes (Voltage: -20/+20 mV) are superimposed at the bottom. (**c**) There is no significant difference (*p* = 0.168; ns: not significant) between the average number of spikes/s of control and the response to air particle movement stimuli. The grey zone around the trendline (slope = 0.0012) is a 95% confidence interval. Data recorded at 15,152 samples/s [*Note*: Different colors are assigned to different waveforms and are consistent throughout the figure]

**Fig. 3:**
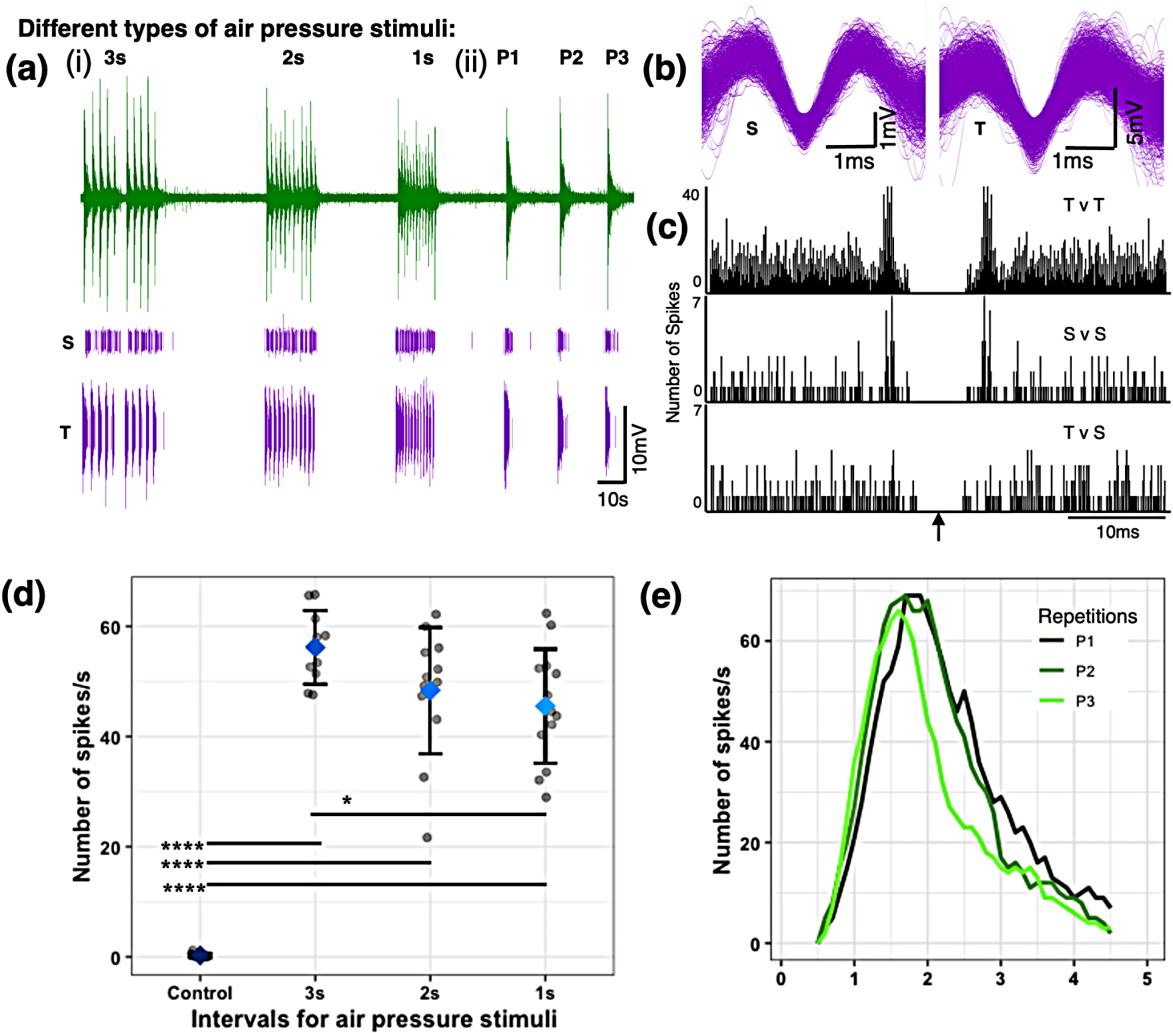
Phasic mechanosensory response to air pressure stimuli (Animal B, Sensillum location: Left Tibia). (**a**) Sample electrophysiological responses from an elongated sensillum to short bursts of forceful breath separated by (i) 3s, 2s, and 1s intervals and (ii) 4 repetitions of prolonged forceful breaths (P1-P3). The upper trace shows the raw record while the shorter (S) amplitude spikes (superimposed in (b, left), Voltage: -2.5/+2.5 mV) are isolated in the middle trace and the taller (T) amplitude spikes (superimposed in (**b**, right), Voltage: -9/+7 mV) are isolated in the lower trace. Only response spikes are shown here. (**c**) Autocorrelation between the taller spikes (T v T), autocorrelation between shorter spikes (S v S) and cross-correlation between the taller and shorter spikes (T v S) suggesting that the responses are from the same mechanosensory cell (T v T y-axis is different from S v S and T v S) Arrow indicates origin or zero lag position. (**d**) There are significant differences between the number of spike/s of the control and that of the stimulus with different time intervals (control-3s: ****p < 0.0001, control-2s: ****p < 0.0001, control-1s: ****p < 0.0001). Adaptation of mechanosensory response is observed as the average number of spikes decreases with the decreasing time interval with a significant difference between the 3s and 1s interval stimuli (3s–1s: *p = 0.021). The grey circles show the real data points, the rhombuses show the average, and the error bars are standard deviations. (**e**) Mechanosensory response to prolonged air pressure stimuli shown with a rolling average (1 s bins) for the 3 repetitions. It shows peak response and recovery of the cell due to the stimulus. Data recorded at 15,152 samples/s

To analyze our recordings using Spike 2 version 8 (CED), we first exported our data as smaller files focusing on each individual stimulus or set of stimuli. We then acquired the spike patterns from the software for all analyses. Based on the similarity in spike waveform shape, we color-coded the spikes for each experiment/animal. We sorted shorter and taller spikes (different in voltage range) separately (shown in the axes, Figs. 2b and 3b, SI. 7b, 8b) to capture all the spikes that may have changed shape through sensory adaptation. To determine if the solifuge sensilla could detect our artificial stimuli, we compared the spike patterns of the responses during stimulus presentations to the control periods (no stimuli present). We compared the ‘average number of spikes/s’ (calculated over 30s) between stimuli and control for the air particle movement stimuli.

For air pressure stimuli, we only showed the response spikes without the background spikes. We also performed autocorrelations (correlating the time series of an individual spike type against itself) and cross-correlations (correlating the time series from two different spikes against each other) for the responses to air pressure stimuli to explore whether the response might be from the same cell (Eggermont 1990) (Fig. 3c, SI. 8c). For air pressure stimuli at different intervals, we collected ‘number of spikes/s’ in 1-second bins at the beginning of each air pressure stimulus/response at the specified intervals. We considered the recording in between the sets of stimuli responses as a control (data not shown). For the prolonged air pressure stimulus, we collected the number of spikes/s for one second before the start of the stimulus to four seconds after the start of the stimulus (total 5 seconds in 100ms bins).

#### Statistical analysis

We analyzed the data using R version 4.1.2 (R Core Team 2021). We used the packages tidyverse, ggplot2, rstatix (function, pairwise_t_test) and zoo (function rollmean). We tested to see if there were any trends in the ‘average number of spikes/s’ for the control (no sound playing) and each of the sound frequency stimuli. We considered the ‘average number of spikes/s’ from 10 Hz to 1000 Hz and applied a linear regression trendline. We compared the ‘average number of spikes/s’ for the control and the frequency stimuli with a pairwise t-test.

With the ‘number of spike/s’ collected from Spike2 for the response to stimuli at different intervals, we performed combined pairwise t-tests to compare the responses to the different interval stimuli with the control and with each other. The p-values for the combined pairwise t-test were adjusted by the Holm method. We used a rolling average method (1 second bins) to plot the response to prolonged forceful breath stimuli. The rolling average method is used to find long-term trends that might be hidden by intermittent fluctuations. We also provided the response with the raw data (SI. 9).

### Behavioral responses to mechanical stimuli

We placed the animal in a plastic circular arena on a granite surface (25 cm x 10 cm). At the beginning of each trial, we positioned the solifuge in the middle of the arena inside a smaller circular barrier (10 cm x 5 cm) and let it acclimatize for 30 min. We recorded the experiments in darkness with an infrared camera with a “night-shot” function (Sony DCR-TRV 351). The darkness and granite substrate removed the possibility of animals responding to visual or substrate-borne stimuli. The surface was cleaned in between trials with ethanol. We observed the occurrence and latency of response behavior after applying the stimuli. The behavior we scored included: (a) Flight behavior, the individual moves away from the stimulus; (b) Confrontation behavior, the individual moves or turns in the direction of the stimulus; (c) Freezing behavior, the individual is in motion and stops after the stimulus; (d) Startle behavior, the individual slightly moves one of its appendages (following prior research by Cloudsley-Thompson 1961; Punzo 1998). We conducted the behavioral response experiments on two consecutive days (1st and 2nd iterations) for each animal with ~ 24 h of rest to determine the consistency in their behavioral responses.

The air particle movement and air pressure stimuli were the same as in the elongated sensilla deflection protocol. We also used tactile stimuli where we (author FC) touched the elongated sensilla on the 4th leg directly using an entomological pin to observe the behavioral responses of the solifuge. We also touched other sensilla on the pedipalps as a control to compare the behavioral responses previously observed. We recorded all interactions with the individual solifuges to verify that the stimulus was applied correctly to the elongated sensilla.

## Results

### Elongate sensilla deflection

We found no discernible deflection of the elongated sensilla on the 4th leg with air particle movement stimuli (all frequencies in low or medium amplitude) when the speaker was placed 10 cm away. Analysing the high-speed camera video, we observed a deflection movement of the sensilla on the 4th leg within the frequency range of 60–100 Hz when a high amplitude (100–105 dB) was used. We also observed deflection of the elongated sensilla under air pressure stimuli produced by “forceful breaths”, and “forced airflows” with a syringe at 20 cm.

### Electrophysiology

#### Air particle movement stimuli

For animal A, when exposed to sound frequency stimuli ranging from 10 to 1000 Hz (shown representative Control, 10 Hz, 50 Hz, 100 Hz, 500 Hz, 1000 Hz), there was no difference in the spike patterns found in the control compared to that in response to the stimuli (Fig. 2a). For the Control traces, we chose a representative control segment that shows all the spikes that appeared throughout the recordings. There were four representative shorter spikes (Fig. 2a top of each) and two taller spikes (Fig. 2a bottom of each) interspersed throughout the recording. While some spikes were more common than others at different frequencies (10 Hz, 50 Hz, 100 Hz, 500 Hz, 1000 Hz), overall, we did not see any differences in spike patterns between the control and air particle movement stimuli. When we analyzed the average number of spikes/s, there was no significant difference between the control and the air particle movement stimuli (pairwise t-test statistic = 1.39, df = 72.34, *p* = 0.168; Fig. 2c). There was also no trend (slope = 0.0012) in the ‘average number of spikes/s’ with increasing frequency. By examining the spike patterns of other solifuges, we found the same results - they did not systematically respond to any of the sound frequency stimuli (SI. 7, Animal B). The lack of systematic response to any of our stimuli suggests that these sensilla are not responsive to the air particle movement produced by low frequencies of sound.

#### Air pressure stimuli

For Animal B, when exposed to air pressure stimuli, we observed a clear phasic mechanosensory response (Fig. 3a). There was a quick response to stimuli which decreased and stopped when exposed to continuous stimulation (P1-P3, Fig. 3a). The superimposed spikes show a similar pattern (shorter (S): Fig. 3b left, taller (T): Fig. 3b right). Another example from a different sensillum of the same animal also shows a similar response (SI. 8a, b).

We ran autocorrelations within the taller spikes (Fig. 3c, top trace: T v T) and within the shorter spikes (Fig. 3c, middle trace: S v S). The clearing around the origins (shown by an arrow) shows no other spike appearing at the same instant as focal spikes, suggesting that all the taller spikes are from the same cell, and all the shorter spikes are from the same cell (owing to the cell’s refractory period). Next, we ran a cross-correlation between the taller and shorter spikes (Fig. 3c, bottom trace: T v S) and again we observed a clearing around the origin or zero lag position (arrow) which also suggests that the taller and shorter spikes are all from the same mechanosensory cell. Such a cell seems likely sensitive to air pressure, with the difference in the waveform of the spikes likely due to changes in ionic concentrations across the cell’s membrane with prolonged mechanical stimulation as the shorter spikes show up after the taller spikes. We see similar results for a different sensillum on the same individual (SI. 8c).

When analyzing the ‘number of spikes/s’ at the beginning of each stimulus/response, we observed an adaptation to the air pressure stimuli. Along with significant differences between the control and the responses at the different time intervals (control-3s: pairwise t-test statistic = -26.44, df = 9.07, *p* < 0.0001; control-2s: pairwise t-test statistic = -14.52, df = 11.03, *p* < 0.0001; control-1s: pairwise t-test statistic = -15.8, df = 12.05, *p* < 0.0001), we observed a significant difference in the ‘number of spikes/s’ between the 3s and 1s interval responses (pairwise t-test statistic = 2.99, df = 20.51, *p* = 0.021) (Fig. 3d). Thus, when the stimuli were applied at very short intervals (1s), the cell’s response exhibited adaptation showing reduced spikes. This suggests that the recovery period of this cell is between 1s and 3s and that this cell is a quiescent cell that is fast adapting and fast recovering in response to the stimuli. The results from other sensilla also showed similar responses (SI. 8d, same individual different sensillum), however, we did not see a significant difference between the ‘number of spikes/s’ of the 3s and 1s intervals.

When exposed to a prolonged air pressure stimulus, we again observed a decrease in spike amplitude (Fig. 3a, e). The response to prolonged stimuli peaks quickly and subsides within 4 s from the start of the stimuli (Fig. 3e). The raw responses (SI. 9a) also show a similar pattern of peaking and subsiding in ~ 4 s. The records from a different sensillum on the same solifuge showed a similar response pattern (SI. 8e, 9b). In summary, the strong responses to forceful breath stimuli suggest mechanosensory properties of the elongated sensilla that is responsive to air pressure stimuli.

**Fig. 4:**
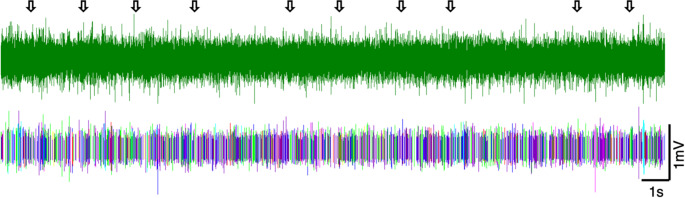
Lack of response to passive breath stimuli (humidity, temperature, and olfaction control for air pressure stimuli) (Animal B, Sensillum location: Right Tibia). The 10 arrows on the electrophysiological recording indicate when the stimulus was applied followed by sorted spikes. There is no response to the stimuli as evidenced by the lack of change in the spike pattern. Data recorded at 15,152 samples/s

We found no evidence of a response to our passive breath stimulus. The arrows on the raw recording and corresponding sorted spikes (Animal B, Fig. 4) show the instances when passive breath stimuli were used. We observed no change in spike activity around those stimulations. Another set of examples from the same sensillum also shows no response to the passive breath stimuli (SI. 10). The lack of response to passive breaths suggests that these sensilla did not respond to any temperature, humidity, or aromatic changes during our stimuli presentations.

To summarize, we observed mechanosensory electrophysiological responses from the elongated sensilla on the 4th pair of legs of solifuges that are responsive to air pressure stimuli but were not responsive to air particle movement stimuli at low frequencies (10–1000 Hz) or to passive breath stimuli. This suggests that these elongated sensilla have a mechanosensory function but are not sensitive enough to detect air particle movement.

### Behavioral responses to mechanical stimuli

We did not find any discernible behavioral responses to any of the frequencies during the low amplitude air particle movement stimuli in either iteration for the tested animals. We did, however, observe some isolated behaviors. For example, one solifuge (Animal 1) walked while moving its pedipalps for 106 s after the 900 Hz stimulus in the 1st iteration. However, this response was not reproducible in the second iteration. During the medium and high amplitude stimuli, we also did not observe any discernible behavioral responses to any of the frequencies played.

Regarding the air pressure stimuli produced by both “forceful breaths” and “forced airflows”, we observed a startle response with a subtle pedipalp movement in both solifuges (Animals 1 & 2). Animal 1 also walked for 3s at the time of stimulation with the “forceful breath” on the 2nd iteration. However, there is no evidence that the individual is actually moving away from the stimulus, and therefore this was not classified as flight behavior. In addition, freezing behavior could not be assessed as the animals always remained motionless when the stimulus was applied. After applying tactile stimuli to the elongated sensilla on the 4th leg, we observed that solifuges performed the confrontation behavior with a latency of less than 1s and moved their pedipalps or chelicerae for 3 to 27 s. The turn was often accompanied by a back-and-forth movement, body shaking and once walking for 23s (Animal 1, 2nd iteration). In the case of the pedipalp sensilla stimulation, we found a response of forward extension of the body with chelicerae movement for 2 s and walking for 24 s immediately after touching these sensilla.

In summary, our behavioral data do not reveal a discernible pattern in solifuges in response to low or high amplitude air particle movement stimuli applied to the elongated sensilla of the 4th leg. However, we did observe isolated behaviors following the application of air pressure and tactile stimuli, such as movements of the pedipalps, walking, and confrontational behavior, among others. This provides support for the mechanoreceptor function of this sensilla.

## Discussion

Our electrophysiological results and sensilla deflection observations suggest that solifuges do not use the elongated sensilla on their 4th pair of walking legs to receive air particle movement signals, but that they can use these sensilla to detect air pressure and other tactile stimuli. Despite their superficially similar appearance to trichobothria (i.e., they are long and thin), these sensilla on solifuges did not respond to low-frequency air particle movement stimuli (10–1000 Hz) and did not exhibit deflection in response to this stimulus. Electrophysiological recordings showed no difference in spike patterns in response to air particle movement stimuli compared to the control periods of stimulus absence. Furthermore, there was no specific behavioral response observed to air particle movement stimuli. In contrast, we found electrophysiological responses of the sensilla to air pressure changes generated by blowing and the sensilla also showed a visible deflection to these same stimuli. The electrophysiological response was phasic, i.e., the cell quickly responded to the stimuli but stopped responding in response to prolonged stimuli, and the cell responded in a fast adapting and fast recovering way. With decreasing intervals between the stimuli, the response exhibited adaptation showing a decreased ‘number of spikes/s’, potentially due to decreased extracellular sodium ion concentration with prolonged stimulation (Kobayashi and Irisawa 1964). The recovery period for this cell was between 1 and 3 s. Behaviorally, in response to the air pressure stimuli, solifuges exhibited a startle response, indicated by small movements of their appendages. Ultimately, despite not being as sensitive as trichobothria, these sensilla can still respond to air pressure changes, which may play an important role for these animals. Finally, in response to tactile stimulation of these sensilla, a clear and consistent confrontation response to the stimulus was observed, confirming a mechanosensory function.

One of the primary goals of this study was to determine if solifuges could detect air particle movement, as this has proven to be important for both prey and predator detection in other arachnid groups (Barth 2000, 2002). Despite not possessing true trichobothria (Shultz 1990; Barth and Holler 1999), we wondered if other elongated sensilla in solifuges might have a similar function. We chose to test the most likely candidates– the longest sensilla on the walking legs. With our experimental design, we found no evidence that the solifuges in our experiments could detect air particle movement with these sensilla. In fact, we found no deflection of the elongated sensilla and no clear or consistent behavioral responses to this type of stimulus. However, it is still possible that one or more of the many other sensilla spread across the walking legs can detect air particle movements. Preliminary observations, (SI. 11), indicate that smaller sensilla respond with visible movement to air particle movement stimuli (50 Hz ~ 110 dB) without eliciting any behavioral response but we observed no movement from the elongated sensilla. Future studies are needed to explore the function of additional solifuge sensilla.

We used artificial stimuli for our experiments here. In nature, however, sound frequencies do not appear as pure tones but instead are mixed with noise and other ecological information. Thus, it is possible that the sensilla are more specially tuned to biologically relevant stimuli. This seems unlikely though, as trichobothria of spiders can respond to a broad frequency range of 10–750 Hz (Barth and Holler 1999). Nonetheless, future studies exploring a broader range of stimuli and potentially more natural stimuli could be informative. It is important to note, however, that electrophysiology studies on solifuges are challenging because of the difficulties in obtaining and maintaining live animals, making such studies difficult.

Though we did not find evidence of air particle movement detection, we did find evidence that the elongated sensilla can detect more forceful mechanosensory stimuli, such as that caused by forceful breath-producing changes in air pressure. We also explored the animal’s behavioral response to air pressure stimuli. In line with our electrophysiology results, we observed a startle response, i.e., subtle pedipalp movement, with pedipalp and head movements when exposed to these stimuli. We also found a clear and consistent confrontational response to a tactile stimulus from the elongated sensilla of the 4th pair of walking legs. The response to mechanosensory stimuli supports early observations of these elongated sensilla being responsive to the slightest touch (Grasse 1968). How the animal uses this information, however, remains to be tested.

This study is only the second study (Cushing et al. 2014) to perform electrophysiological recordings on the elusive solifuges and the first on the elongated sensilla of the 4th pair of walking legs. These animals are difficult to capture in large quantities needed for extensive studies (Cushing et al. 2014; Cushing and González-Santillán 2018). It took authors RRJ and EG several days to collect the 12 animals despite using both hand collecting and the light arrays methods. Solifuges are also sensitive to captivity and die easily. Out of the 12 animals, we collected reliable data from only five and unfortunately, these animals were not frozen for post-experiment anatomical analyses. Nonetheless, we are confident that the action potentials we collected came from sensory cells within the elongated sensillum. The photo in SI. 1c is the best evidence for this as the electrode is clearly within the socket of the sensillum. Extracellular signals can only be adequately detected within ~ 50–100 microns of a spiking cell, as they become indistinguishable from background noise at greater distances. Our action potentials had very good signal-to-noise ratios (some > 10:1), making it impossible that those signals could have emanated from a neighboring sensillum.

In future studies, if additional animals could be collected and maintained, it would be good to expand on our results with additional stimuli and additional experiments. Future studies could, for example, use a calibrated particle velocity sensor (e.g., an anemometric microflow device or pressure gradient microphone) positioned next to the sensilla to confirm the stimuli and enable a better estimation of near versus farfield sound stimuli. Given that this exploratory study took advantage of available animals, such equipment was not readily available at the time of experiments. Future studies could also record the response to the mechanical stimuli before and after ablation of the elongated sensilla, and this could be paired with behavioral ablation studies as well. We note, however, that all these studies would be challenging considering the availability of these elusive animals and the fact that they are often uncooperative in an experimental setting.

The detection of air particle movement using trichobothria is important for capturing prey in spiders (Barth 2000) or escaping predators in other arthropods (Tautz and Markl 1978; Ashford et al. 2018). Recent studies also suggested the use of trichobothria in receiving air particle movement courtship signals (Rundus et al. 2010; Choi et al. 2019; Kundu et al. 2022). However, since solifuges are fast-moving (Punzo 1998) and live mostly in xeric environments (Schmidt 1993), it is possible that detecting changes in air pressure and not in air particle movement is sufficient for them to receive meaningful biological information from their environment. We suspect that solifuges likely rely on information gathered from chemosensory and mechanosensory sensilla on their pedipalps (Cushing et al. 2014), and possibly on their malleoli (Brownell and Farley 1974), to capture prey. It has also been noted that they capture terrestrial prey with direct contact and that the elongated sensilla plays a role in this prey capture (Bolwig 1952). Thus, air particle movement does not appear important in the foraging success of solifuges. Furthermore, there appears to be little opportunity for solifuges to use air particle movement during reproductive interactions. It has been observed, for example, that many male solifuges dig around the burrows of females, who then appear from their burrows in response to the digging (Wharton 1986). If females are in burrows with males digging above, chemical and more forceful mechanical signals than air particle movement are likely important. Female-male encounters also demonstrate rapid transitions from initial contact to copulation (Rowsell and Cushing 2020), leaving little opportunity for near field communication.

We hypothesize that the main behavioral function of air pressure detection by the elongated sensilla on the 4th pair of walking legs in solifuges is predator avoidance. Solifuges are often predated by larger animals that may produce air pressure changes when moving quickly through the environment (Arlettaz et al. 1995; Anderson et al. 1999). Large birds like raptors, owls, New World shrikes, Old World larks, wagtails, and bustards are known to be natural predators of solifuges (reviewed in Hebets et al. 2023). The response to more forceful mechanosensory stimuli could facilitate rapid escape responses by enabling solifuges to detect the flapping of wings by birds which produce changes in air pressure. Solifuges are also predated on by small mammals and reptiles (reviewed in Hebets et al. 2023). This function could be similar to the function of cerci in crickets, which clearly aid in escape behavior (Boyan and Ball 1990). It is less clear, however, how these mammalian and/or reptilian predators might generate air pressure cues that could elicit solifuge anti-predator responses. All of these functions require testing.

Our behavioral observations suggest that solifuges respond to air pressure stimuli with low-intensity displays like the startle behavior. We did not observe any high-intensity displays (i.e., attack, stridulation, flight) (Cloudsley-Thompson 1961; Punzo 1998). This pattern could be attributed to the possibility that the intensity of our stimulus may not be enough to elicit these alternative types of behavior. Alternatively, or additionally, freezing may be an adaptive response. Further studies are now needed to understand how solifuges respond to natural mechanosensory stimuli such as the beating wings of a bird, the incoming gliding motion of a large predator, or even high-speed wind, possibly during a sandstorm (a possible abiotic condition in many solifuge habitats) (reviewed in Hebets et al. 2023).

We are confident that our electrophysiological responses were due to physical air pressure and not any other stimuli. To confirm that the observed response to our forceful breath stimulus was not due to any changes in temperature, humidity, and/or chemical cues, for example, we used a passive breath stimulus. As expected, we observed no response to the passive breaths. It is worth emphasizing that we conducted exploratory analyses to observe if there was an electrophysiological response from the elongated sensilla to a chemical − 1-hexanol - but we did not find a discernible response (Kundu P. unpublished data). Our study thus provides no evidence of thermo-reception, hygro-reception, or chemoreception by these sensilla, but it cannot yet be ruled out. It is possible, for example, that our passive breath did not alter temperature, humidity, or chemical stimuli across the 23 cm that the breath traveled. Though unlikely, it remains possible that there are other biologically meaningful stimuli, such as particular chemicals, that might elicit a response.

Though this study did not include any formal morphological analyses or exploration such as scanning electron microscopy, we did observe sockets at the base of the elongated sensilla under a light microscope, suggesting a mechanosensory function. Morphological explorations by electron microscopy of the elongated sensilla from the 4th pair of legs in other solifuges species did not reveal the presence of pores (Oviedo-Diego M. and Cargnelutti F. unpublished data) that would suggest a chemosensory function (Foelix 1985), consistent with our findings. Further morphological analyses of hair structure and innervation might, however, provide insight into the potential for chemoreception. Ultimately, our data provide no evidence of anything other than mechanoreception for these elongated sensilla on the 4th pair of walking legs of solifuges.

Now that a mechanosensory function, including the potential detection of air pressure, has been confirmed in solifuges, behavioral studies in field conditions are needed to relate this sensory capacity to meaningful natural stimuli (e.g., emulating potential predators) and thus natural function. While solifuge behavior remains poorly studied (reviewed in Hebets et al. 2023), with little known about natural anti-predator behavior, the knowledge of physiological sensory mechanisms provided in this study provides a foundation for future research.

## Conclusion

We exposed the elongated sensilla on the 4th pair of walking legs of solifuges to artificial air particle movement and air pressure stimuli in an electrophysiology recording setup, and we evaluated in detail the deflection of the sensilla and the behavioral response of the individuals to these stimuli. From the analysis of the electrophysiology and behavioral data, our study suggests that the elongated sensilla on these 4th legs function as mechanoreceptors. Both the sensilla and the animals were unresponsive to air particle movement stimuli but were responsive to stronger air pressure stimuli. Furthermore, the phasic response pattern we saw to mechanosensory stimuli suggests the sensilla contain a fast adapting, fast recovering cell. More experiments exploring chemoreception, hygroreception, thermoregulation, and natural behavioral analysis might provide us with more understanding of the function of these extraordinarily long sensilla in the solifuges’ environment.

## Electronic supplementary material

Below is the link to the electronic supplementary material.

Supplementary Material 1

Supplementary Material 2

Supplementary Material 3

Supplementary Material 4

Supplementary Material 5

Supplementary Material 6

## Data Availability

No datasets were generated or analysed during the current study.
