# Supplementary material for: Electrophysiological and behavioral responses of elongated solifuge sensilla to mechanical stimuli
Source: J Comp Physiol A Neuroethol Sens Neural Behav Physiol. 2025 Feb 5;211(3):277–92. doi: 10.1007/s00359-025-01731-y (PMC12081520; doi:10.1007/s00359-025-01731-y)
Supplement: Supplementary file 1 — Supplementary Material 1 [file 359_2025_1731_MOESM1_ESM.docx]

**Supplementary Information (SI)**


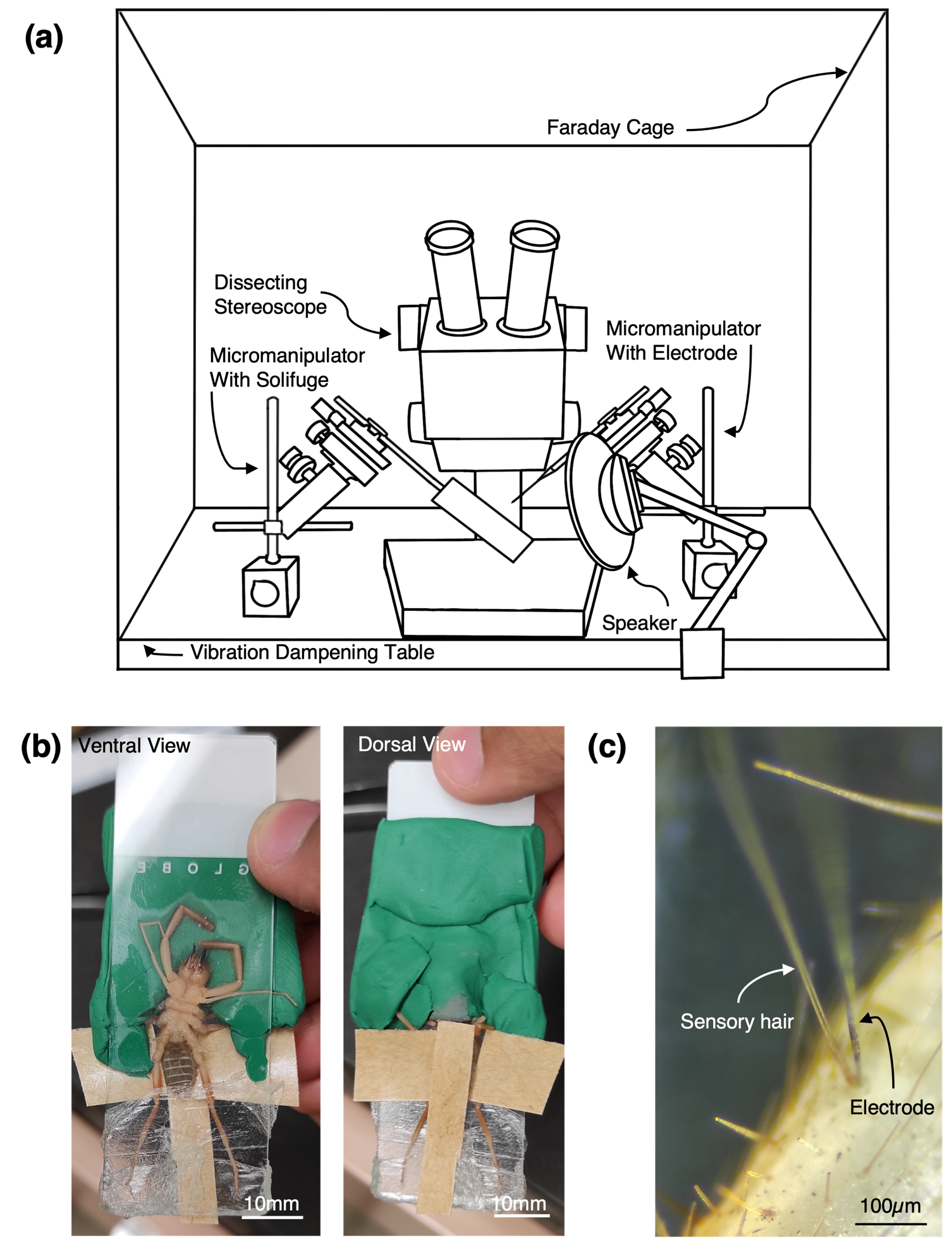


**SI. 1** Electrophysiology recording setup. **(a)** A live solifuge was fixed on a slide, placed on a micromanipulator, and positioned under a dissecting stereoscope inside a Faraday cage. A second micromanipulator was used to maneuver a tungsten recording electrode to the base of a target sensory hair. A speaker was clamped to the Faraday cage and positioned ~10 cm away from the recorded hair. **(b)** A solifuge fixed to a microscope slide with double-sided tape and modeling clay with exposed hindlegs in preparation for electrophysiology (left: ventral view; right: dorsal view). **(c)** Close-up of a solifuge’s 4th leg (left tibia) with an electrode inserted in the base of a sensillum.

**SI. 2** Video showing lack of behavioral response of a female solifuge to air particle movement stimuli. We played the initial frequencies (10Hz, 50Hz, 100-1000Hz at 100Hz increments) in the video at the same output level as 100Hz at ~74dB. We also tested 50Hz at ~110dB. We did not observe any distinct behavioral response.

<https://drive.google.com/file/d/1VfLMfv4ICh798PLHvsGT3ZVt4zEO5r1L/view?usp=sharing>

**SI. 3** Video showing elongated sensilla movement to forceful breath air pressure stimuli.

<https://drive.google.com/file/d/1VLc5_WsZg2zBIhR7K0_lkcIns_x_e7eY/view?usp=drive_link>

**SI. 4** Video showing angle difference (degrees) of elongated sensillum deflection with time as a response to forceful breath stimuli in SI. 3.

<https://drive.google.com/file/d/1Kn8ZJlqgpAFwm03ma7_yOEJGbsOy-vla/view?usp=sharing>

**
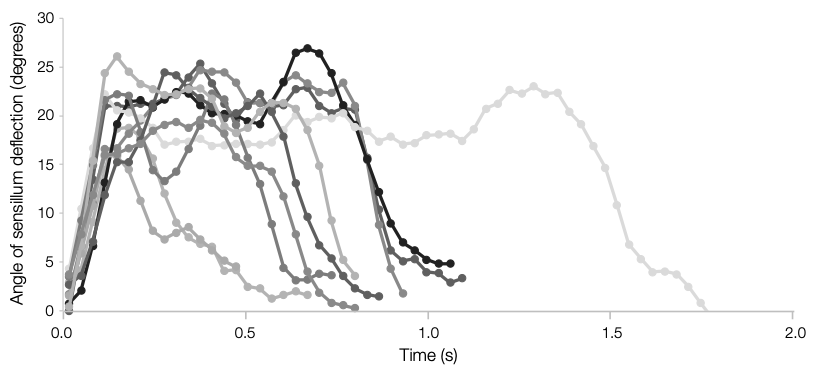
**

**SI. 5** Superimposed responses (angle of deflection (degrees) over time) of the elongated sensillum shown in SI. 3 and SI. 4 to 10 forceful breath stimuli.

**SI. 6** Video showing behavioral responses of a female solifuge to air pressure stimuli. We used forceful breaths and 10cc/20cc syringes to elicit responses. We observed startle, freeze, and escape responses.

<https://drive.google.com/file/d/1VS0TQMYIcBL1eMUqXE6DZQnFIdyD5TSI/view?usp=drive_link>

**
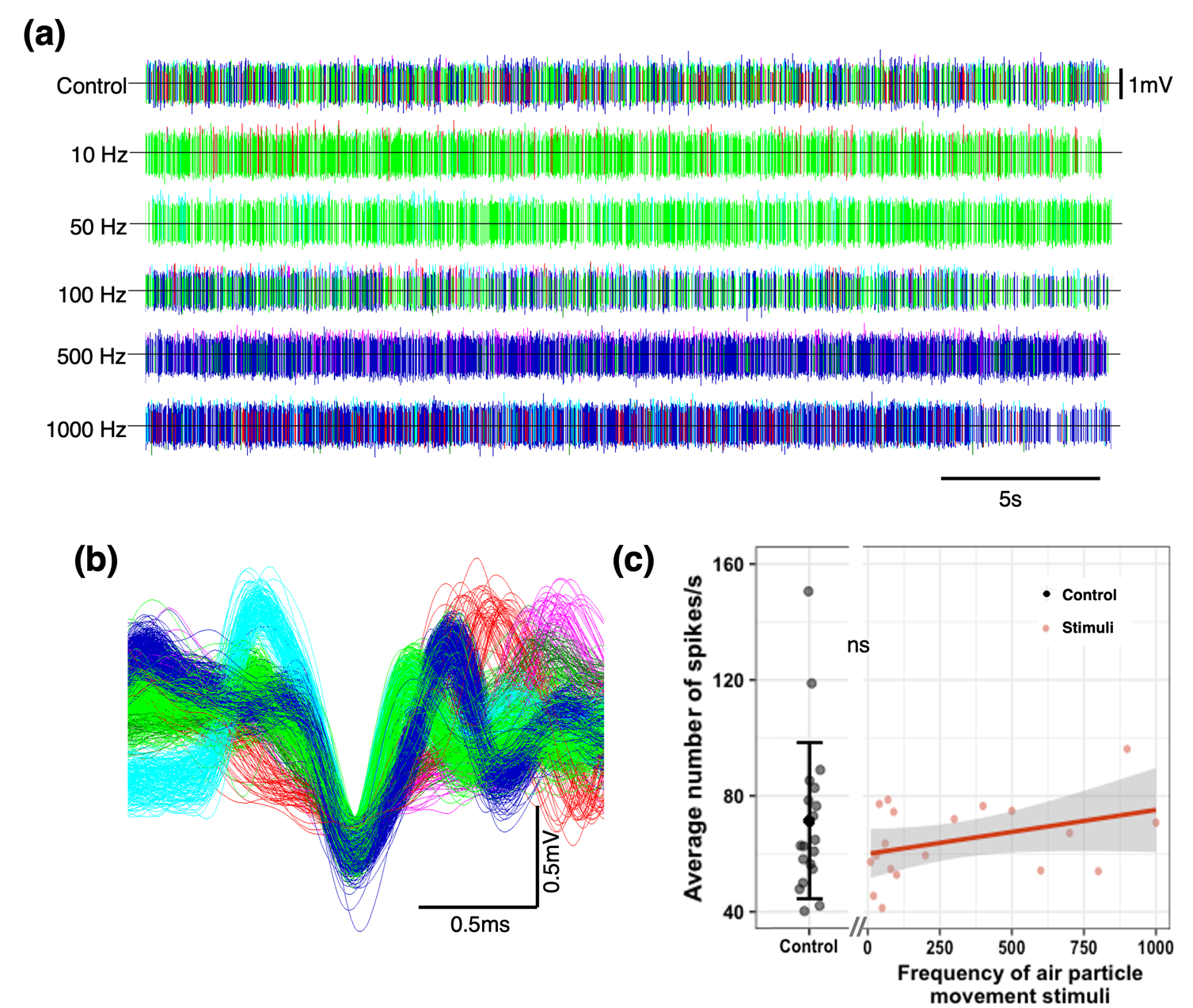
**

**SI. 7** Lack of response to air particle movement stimuli (Animal B, Sensillum location: Left Tibia). **(a)** Representative electrophysiological recordings from a long sensory hair on the tibia of the left 4^th^ leg at Control (No stimulus), 10 Hz, 50 Hz, 100 Hz, 500 Hz and 1000 Hz (stimuli). The traces are the sorted spikes for each. Notice that there are no significantly different spikes appearing between the control and stimulus and the difference in axes scales. **(b)** The spikes are superimposed. **(c)** There is no significant difference (pairwise t-test statistic = 0.943, df = 26.479, p=0.354; ns: not significant) between the average number of spikes/s of control and the response to air particle movement stimuli. The grey zone around the trendline (slope=0.015) is a 95% confidence interval. Data recorded at 15,152 samples/s. [Note: Different colors are assigned to different waveforms and are consistent throughout the figure]


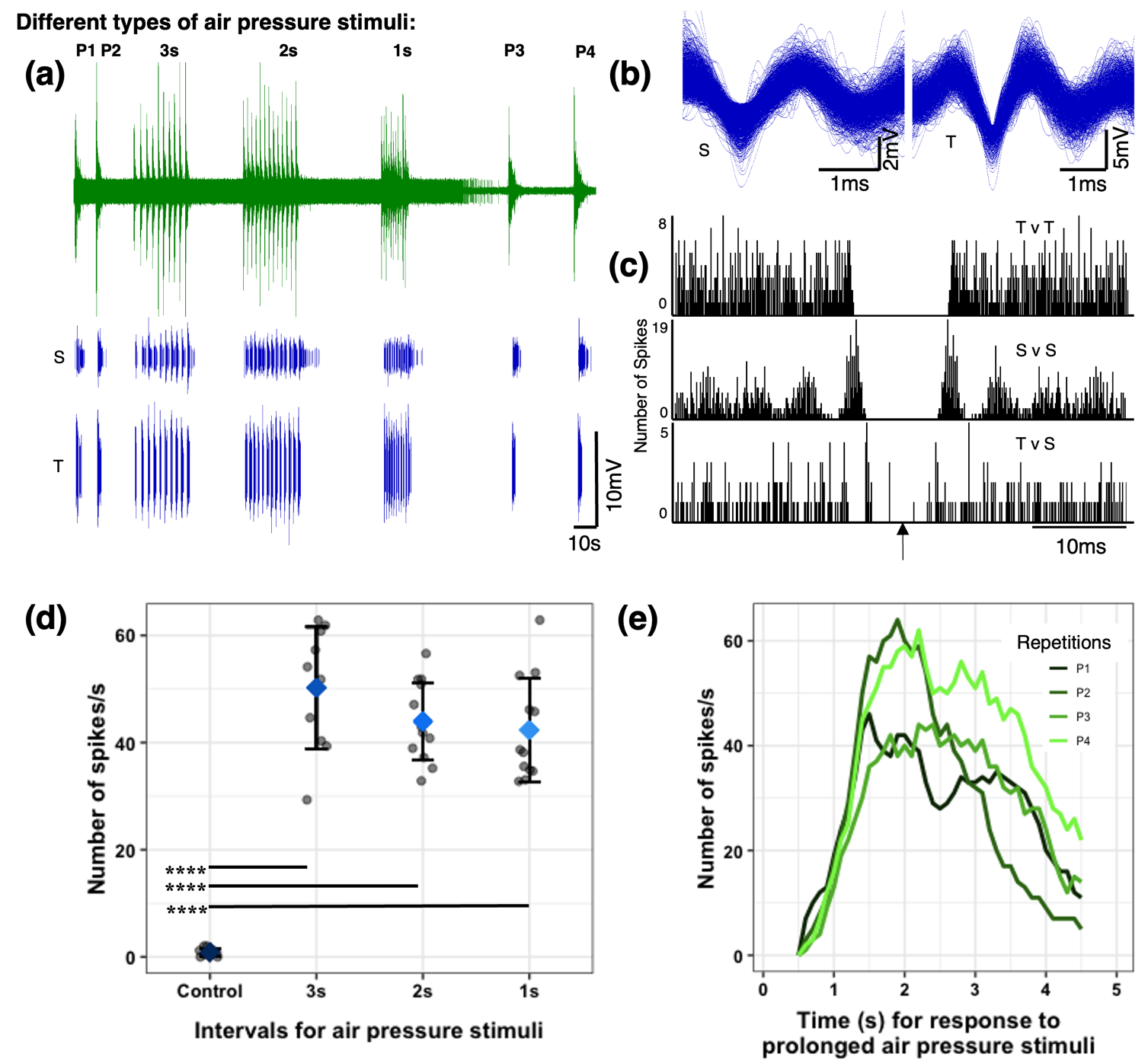


**SI. 8** Phasic mechanosensory response to air pressure stimuli (Animal B, Sensillum location: Right Tibia). **(a)** Sample electrophysiological responses from an elongated sensillum to short bursts of forceful breath separated by 3s, 2s, and 1s intervals and 4 repetitions of prolonged forceful breaths (P1-P4). The upper trace shows the raw record while the shorter (S) amplitude spikes (superimposed in (**b**, left)) are isolated in the middle trace and the taller (T) amplitude spikes (superimposed in (**b**, right)) are isolated in the lower trace. Only response spikes are shown here. **(c)** Autocorrelation between the taller spikes (T v T), autocorrelation between shorter spikes (S v S), and cross-correlation between the taller and shorter spikes (T v S) suggesting that the responses are from the same mechanosensory cell. Arrow indicates the origin or zero lag position. **(d)** There are significant differences between the number of spike/s of the control and that of the stimulus with different time intervals (control-3s: ****p<0.0001, control-2s: ****p<0.0001, control-1s: ****p<0.0001). Even without a significant difference between the responses at 3s and 1s intervals, we can observe that the average at 1s intervals is lower than the 3s intervals. The grey circles show the real data points, the rhombuses show the average, and the error bars are standard deviations. **(e)** Mechanosensory response to prolonged air pressure stimuli shown with a rolling average (1 second bins) for the 4 repetitions. It shows peak response and recovery of the cell due to the stimulus. Data recorded at 15,152 samples/s.


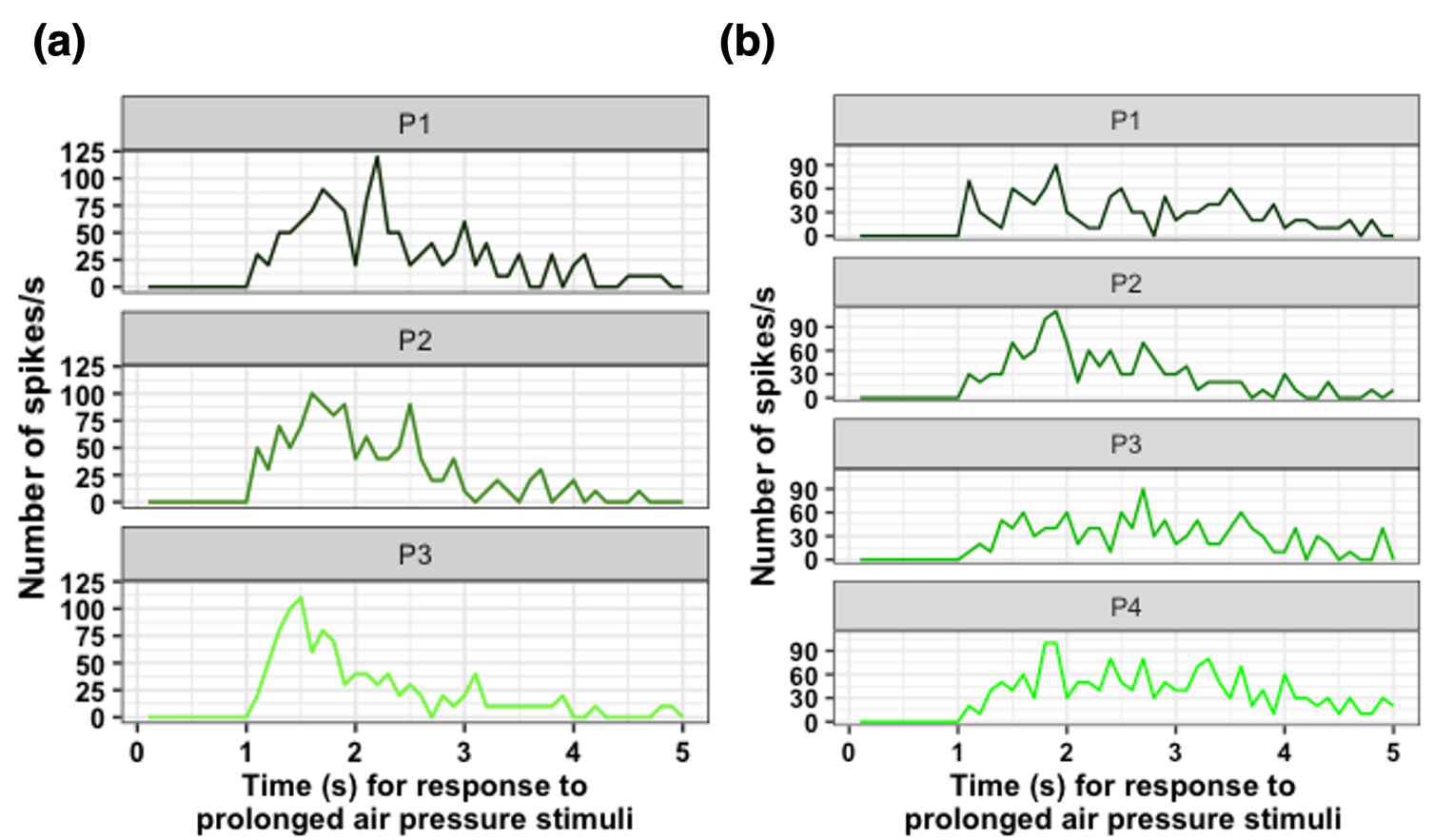


**SI. 9** Raw data for responses to prolonged air pressure stimuli (Animal B). **(a)** The data of 3 repetitions of responses to prolonged air pressure stimuli from left tibia sensillum. **(b)** The data for 4 repetitions of responses to prolonged stimuli from right tibia sensillum. Data recorded at 15,152 samples/s.


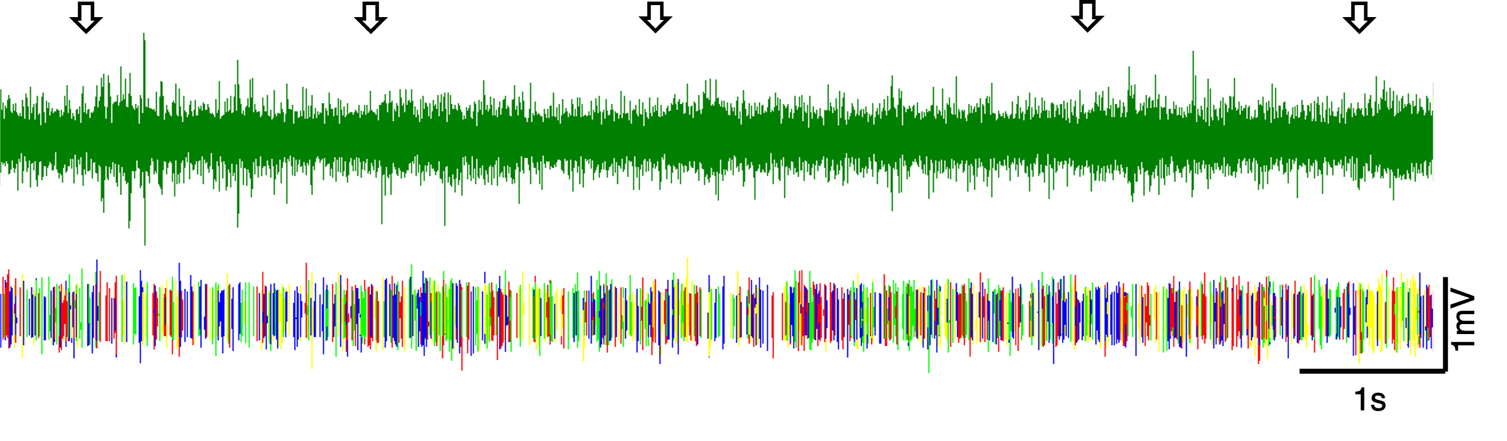


**SI. 10** Lack of response to passive breath stimuli (humidity, temperature, and olfaction control for air pressure stimuli) (Animal B, Sensillum location: Right Tibia). The 5 arrows on the electrophysiological recording indicate when the stimulus was applied followed by sorted spikes. There is no response to the stimuli as evidenced by the lack of change in the spike pattern. Data recorded at 15,152 samples/s.

**SI. 11** Video showing smaller sensilla movement in response to 50Hz air particle movement stimuli at ~110dB

<https://drive.google.com/file/d/1Vqbt33ExQjjQwe4_cdRiaVH__Flik0mo/view?usp=sharing>
